# Supplementary material for: A novel source of arterial valve cells linked to bicuspid aortic valve without raphe in mice
Source: eLife. 2018 Jun 29;7:e34110. doi: 10.7554/eLife.34110 (PMC6025960; doi:10.7554/eLife.34110)
Supplement: Transparent reporting form [file elife-34110-transrepform.docx]

***eLife’s* transparent reporting form**

We encourage authors to provide detailed information *within their submission* to facilitate the interpretation and replication of experiments. Authors can upload supporting documentation to indicate the use of appropriate reporting guidelines for health-related research (see [EQUATOR Network](http://www.equator-network.org/%20)), life science research (see the [BioSharing Information Resource](https://biosharing.org/)), or the [ARRIVE guidelines](http://www.plosbiology.org/article/info:doi/10.1371/journal.pbio.1000412) for reporting work involving animal research. Where applicable, authors should refer to any relevant reporting standards documents in this form.

If you have any questions, please consult our Journal Policies and/or contact us: [editorial@elifesciences.org](mailto:editorial@elifesciences.org).

**Sample-size estimation**

- You should state whether an appropriate sample size was computed when the study was being designed
- You should state the statistical method of sample size computation and any required assumptions
- If no explicit power analysis was used, you should describe how you decided what sample (replicate) size (number) to use

Please outline where this information can be found within the submission (e.g., sections or figure legends), or explain why this information doesn’t apply to your submission:

In this developmental biology study embryos have been used as the individual experimental unit (n). In the majority of cases the data is qualitative rather than quantitative and thus sample size estimation was not carried out. In the experiment to determine the proportion of each population of progenitor cell in the developing valve leaflets – presented in Figure 3) we were comparing data to a previous published study (Phillips et al, 2013 - reference 7) and thus matched the sample numbers to this previous study.

**Replicates**

- You should report how often each experiment was performed
- You should include a definition of biological versus technical replication
- The data obtained should be provided and sufficient information should be provided to indicate the number of independent biological and/or technical replicates
- If you encountered any outliers, you should describe how these were handled
- Criteria for exclusion/inclusion of data should be clearly stated
- High-throughput sequence data should be uploaded before submission, with a private link for reviewers provided (these are available from both GEO and ArrayExpress)

Please outline where this information can be found within the submission (e.g., sections or figure legends), or explain why this information doesn’t apply to your submission:

In each case a minimum of three embryos was utilized for each experimental stage, orientation of histological section, and assay (for example different antibody). These analyses were carried out as separate experiments on different occasions. These were the biological replicates. In addition, multiple histological sections (in most cases > 10) from each embryo were examined for consistency; these were the technical replicates. This is standard procedure in the field. As the data was generally consistent between developmental stages and between the different orientations of histological section, this gives additional confidence to the data obtained. This information can be found in the Materials and Methods section and in the relevant Figure legends. All data was included in analyses with no outliers excluded.

**Statistical reporting**

- Statistical analysis methods should be described and justified
- Raw data should be presented in figures whenever informative to do so (typically when N per group is less than 10)
- For each experiment, you should identify the statistical tests used, exact values of N, definitions of center, methods of multiple test correction, and dispersion and precision measures (e.g., mean, median, SD, SEM, confidence intervals; and, for the major substantive results, a measure of effect size (e.g., Pearson's r, Cohen's d)
- Report exact p-values wherever possible alongside the summary statistics and 95% confidence intervals. These should be reported for all key questions and not only when the p-value is less than 0.05.

Please outline where this information can be found within the submission (e.g., sections or figure legends), or explain why this information doesn’t apply to your submission:

Chi squared analysis was used to evaluating the proportions of each progenitor cell population within developing valve leaflets – presented in Figure 3.

Post hoc pairwise chi-squared comparison between the primordia, with Bonferroni correction for multiple testing, confirmed that the aortic non-coronary primordium was statistically different from all other primordia. This information can be found in the Materials and Methods section and within the relevant section of text in the Results.

(For large datasets, or papers with a very large number of statistical tests, you may upload a single table file with tests, Ns, etc., with reference to sections in the manuscript.)

**Group allocation**

- Indicate how samples were allocated into experimental groups (in the case of clinical studies, please specify allocation to treatment method); if randomization was used, please also state if restricted randomization was applied
- Indicate if masking was used during group allocation, data collection and/or data analysis

Please outline where this information can be found within the submission (e.g., sections or figure legends), or explain why this information doesn’t apply to your submission:

Groups were assigned strictly on the basis of genotype and developmental stage (corroborated where possible by somite counting and assessment of other external features such as limb maturity) so that embryos for comparison were stage matched.

**Additional data files (“source data”)**

- We encourage you to upload relevant additional data files, such as numerical data that are represented as a graph in a figure, or as a summary table
- Where provided, these should be in the most useful format, and they can be uploaded as “Source data” files linked to a main figure or table
- Include model definition files including the full list of parameters used
- Include code used for data analysis (e.g., R, MatLab)
- Avoid stating that data files are “available upon request”

Please indicate the figures or tables for which source data files have been provided:

Quantitative data is uploaded for Figure 3 E,F and J. These are provided as an Excel file.
